# Datasets on the mechanical behavior of rotary-cut veneer under tensile, compressive, and shear loading

**DOI:** 10.1038/s41597-026-07687-1

**Published:** 2026-06-26

**Authors:** Robert Krüger, Beate Buchelt, Mario Zauer, André Wagenführ

**Affiliations:** TUD Dresden University of Technology, Institute of Natural Materials Technology, Dresden, Germany

## Abstract

In order to improve the predictability of the mechanical behavior of veneer composite materials, such as plywood or laminated veneer lumber, calculations and simulations require material data for their single layers (veneer). Until now, two factors have prevented this from being achieved. First, complete material parameters and raw data describing the mechanical behavior of rotary-cut veneer are unavailable in the literature. Second, there are no standardized or suitable test methods for determining the necessary mechanical properties. This article presents suitable test methods for determining the mechanical behavior of rotary-cut veneer under tensile, compressive, and shear loads, as well as the resulting material data sets. These data sets, obtained from rotary-cut veneer of European beech and birch, are available in the Zenodo open data repository.

## Background & Summary

Rotary-cut veneer is widely used in the production of veneer composites, a specific type of wood composite that uses veneer as a single layer. These composites can be used and optimized in the construction of technical components through purposeful dimensioning. Optimization is achieved through the orientation and arrangement of the single layers (veneers) considering the subsequent stress. A stress-appropriate design of a laminated material requires the mechanical properties of the veneers used as single layers. Thus, the mechanical parameters of veneers for the fundamental stresses, such as tension, compression, and shear, are necessary to predict the mechanical properties of veneer composites. Currently, only a few parameters are available in the literature, primarily the modulus of elasticity and tensile strength parallel to the fiber direction^1–7^. However, there are currently no datasets describing the complete mechanical behavior of rotary-cut veneer. Complete material datasets are necessary in materials science to enable big data approaches to material discovery, characterization, and modeling^8,9^.

Veneer, as a technical product made from the natural material wood, has a complex anatomical material structure with orthotropic material behavior. Therefore, there are two material directions, parallel to fiber and perpendicular to fiber, which must be taken into account when determining the material properties. In addition, there are a number of other factors that influence the mechanical properties, such as climatic conditions or material density.

Table 1 shows all mechanical parameters necessary for a complete veneer characterization.

**Table 1:** Essential parameters for a material model based on veneer.

Stress	Engineering constant	Failure strain	Strength
Tension	**E**_**00**_; E_90_; µ	**ε**_00_; **ε**_90_	**σ**_**00**_; σ_90_
Compression	E_00_; E_90_; µ	**ε**_00_; **ε**_90_	σ_00_; σ_90_
Shear	**G**	**γ**	**τ**

Available parameters are in bold.

E … Young´s modulus; µ … Poisson’s ratio; ε … strain; σ … strength; G … shear modulus; γ … shear strain; τ … shear strength; 00 … parallel to fiber; 90 … perpendicular to fiber.

In practice, single veneers are rarely used for technical applications. Instead, veneer composites are formed by arranging veneer layers in a specific laminate structure and bonding them together with adhesive. Because veneer is porous, adhesive infiltrates the material structure during production, creating a transition zone at the veneer border. Therefore, it can be assumed that this transition zone affects the mechanical properties of the single veneer layers.

Adequate testing methods are essential for determining material properties under tensile, compressive, and shear stress. Currently, there are no standardized methods for determining veneer material properties. Thus, the research projects “Wood-based Materials in Mechanical Engineering: Calculation Concepts, Parameter Requirements, and Parameter Determination” (HoMaba)^10^ and “Material and Technology Development for Flame-Retardant Veneer-Basalt Fiber Composites” (HoBaCo)^11^ adapted or developed suitable measurement methods for characterizing veneer materials based on methods available for solid wood^12–14^. These methods were used to determine complete datasets for rotary-cut veneers of European beech and birch. Some of the material data determined has been published and discussed in various publications^12–15^.

In line with the growing scientific culture of making data comprehensive, discoverable, accessible, interoperable, and reusable (FAIR), the determined material properties, including their measured stress-strain curves, were made available as raw data in repositories^16,17^.

## Methods

This section first describes the materials used to determine material properties. The veneers used, their origin, and their processing differ depending on whether they are from the HoMaba or HoBaCo research projects; therefore, they are described separately. Next is a description of the test methods used under tensile, compressive, and shear loads. These methods are the same for all materials.

### Material

For the HoMaba project, European beech (Fagus sylvatica L.) and birch (Betula pendula Roth.) were used in the investigations. The Eberswalde University for Sustainable Development (Germany), a project partner, was responsible for sourcing the tree trunks. The material was taken from the trunks of trees in a single habitat (Germany; GPS location 52.89619480645926, 13.995167324170996) with comparable growing conditions. The trees were spaced between 2 and 100 m apart. The trunks had a minimum length of 4 m, so each trunk was cut into two 2-meter-long sections. The sections were mixed in a crisscross configuration to produce veneers. Fraunhofer Institute for Wood Research WKI (Germany), another project partner, was responsible for peeling the trunk sections into veneers. Before veneer production, the beech logs were steamed at 70 °C for 72 h in an electrically heated, pressureless steaming chamber. The birch logs could be peeled straight from the tree without pretreatment. Rotary-cut veneers of different thicknesses (1, 2, and 3 mm) were produced in a laboratory environment using a laboratory machine (Raute Corporation, Nastola, Finland). The veneers were then dried at a temperature of 60 °C for 48 h.

As described earlier, it can be assumed that the adhesive used affects the material properties of the individual veneer layers in a veneer composite. For this reason, half of the veneers were coated with adhesive in order to investigate this effect. They were coated on both sides with a phenol-resorcin-formaldehyde resin adhesive (Aerodux 185, Dynea, Lillestrøm, Norway). The adhesive was applied at a quantity of 220 g/m^2^ to each veneer surface. The veneers were pressed in a laboratory press at 90 °C with 10 bar pressure for 120 s. Material tests with these veneer materials were conducted at a climate of 20 °C/50% relative humidity. This test climate was established as standard climate in other studies for mechanical engineering applications^18^.

For the HoBaCo project, industrially produced 2.5-mm-thick rotary-cut veneers made of European beech (Fagus sylvatica L.) were used. These veneers were sourced from Röchling Industrial SE & Co. KG in Haren, Germany. Half of the veneers were coated on both sides. The veneers were dried in a belt dryer at 190 °C to a moisture content of ~0.5% and then coated with a bio-phenolic resin developed in the laboratory (Fraunhofer Institute for Applied Polymer Research IAP, Germany). The resin was applied manually with an application roller at 120 g/m^2^. The veneers were pressed in a laboratory press at 140 °C with 10 bar pressure for 2 h. The internal data sheet for the laboratory bio-phenolic resin used indicates the very long pressing time compared to the HoMaba samples. Material tests with these veneer materials were conducted in climates of 20 °C/50% relative humidity and 20 °C/85% relative humidity, respectively.

All test samples were cut out of the veneers using a CO2 laser. The goal was to obtain 20 valid results for each testing method and series using 25 prepared samples.

### Tension test

**Fig. 1:**
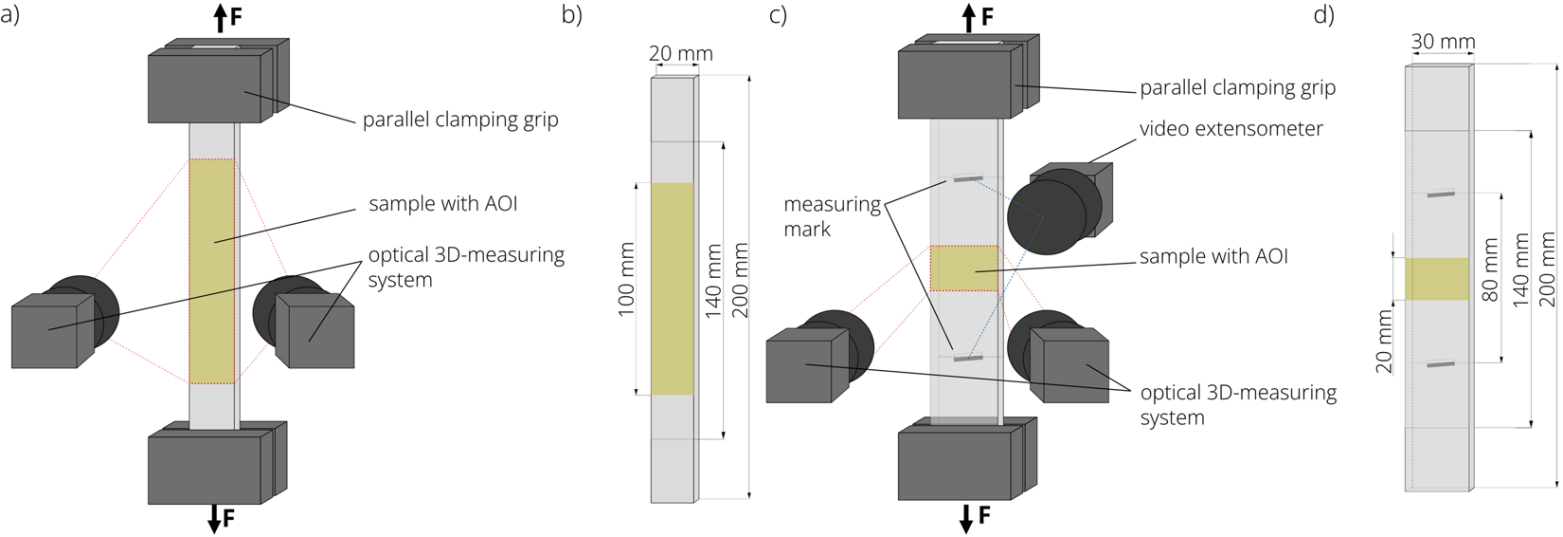
Tension testing method; (**a**) test setup for 00-direction; (**b**) sample dimension for 00-direction; (**c**) test setup for 90-direction; (**d**) sample dimension for 90-direction; AOI – area of interest.

Tensile tests were performed parallel (00-direction) and perpendicular (90-direction) to the fiber direction using a universal testing machine (Inspekt 10, load capacity 10 kN, Hegewald & Peschke, Nossen, Germany). Figure 1 shows the test setups for the 00 and 90-direction, as well as the sample dimension used for the corresponding tests.

The rectangular test specimens had a length of 200 mm in the loading direction. The width was 20 mm for the 00-direction specimens and 30 mm for the 90-direction specimens (see Fig. 1b,d). The greater width of the 90-direction specimens was necessary to ensure sufficient measurement accuracy for the transverse strain in that direction. The samples were clamped using parallel clamping grids, leaving a clear test length of 140 mm between the grips. Measurements were performed in a strain-controlled manner at a strain rate of 1% per minute.

A stereo camera system (Aramis Adjustable 12 M, Carl Zeiss GOM Metrology, Braunschweig, Germany), which applies the principles of digital image correlation (DIC), was used for the strain measurement. A contrast speckle pattern was applied to the area of interest (AOI) on the closed side of the veneer surface. First, the samples were primed with white paint using a roller within the AOI. After drying, stamp-like speckles were sprayed on using a black paint airbrush. For tests in the 00-direction, strain in the loading direction was measured over a 100 mm length, while transverse strain was measured according to sample width. For the tests perpendicular to the fiber direction (90 direction), the GOM system was only used to measure transverse strain. A smaller measuring volume with a higher resolution was used to increase the accuracy of the transverse strain measurement due to the very low strain values in this test direction. Therefore, the camera system could only measure the longitudinal strain of the test specimen over a maximum length of 20 mm. However, this does not correspond to a global strain measurement over a large section within the free clamping length of the test specimen. For this reason, a video extensometer was also used to determine the strain in load direction over an 80 mm range on the back of the specimen using measuring marks. Detailed information on the DIC setup used and its parameters is provided in Table 2.

**Table 2:** Parameters used for DIC in dependence of testing method, based on the specifications in^19^.

Test method	Tension		Compression		Shear
Grain direction	00	90	00	90	—
Camera	Aramis Adjustable 12 M, Carl Zeiss GOM Metrology, Braunschweig, Germany				
Image Size	12Mpx, 4096 × 3000 px^2^				
Lens	Titanar B 75 mm				
Focal Length	MV180	MV35	MV35	MV35	MV35
Aperture	f/16	f/22	f/22	f/22	f/22
Field-of-View	180 × 130 mm^2^	35 × 25 mm^2^	32 × 25 mm^2^	35 × 25 mm^2^	35 × 25 mm^2^
Depth-of-Field	130 mm	10 mm	10 mm	10 mm	10 mm
Image Scale	22.76 px/mm	117 px/mm	117 px/mm	117 px/mm	117 px/mm
Stereo Angle	25°				
Stand-Off Distance	1045 mm	345 mm	345 mm	345 mm	345 mm
Image Acquisition Rate	1 Hz	1 Hz	1–2 Hz	0.5–1 Hz	0.333–1 Hz
Patterning Technique	white primer applied with a roller and black speckles added with an airbrush				
Subset Size	16 px	60 px	60 px	60 px	15 px
Step Size	13 px	40 px	40 px	40 px	10 px

### Compression test

**Fig. 2:**
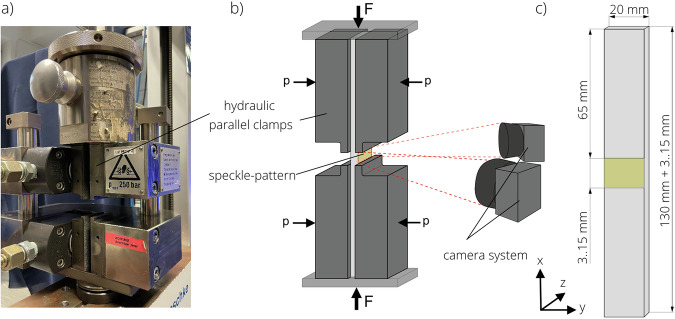
Compression test method; (**a**) testing device; (**b**) test setup; (**c**) sample dimension.

Figure 2 shows the test setup for the compression test. The testing device consists of a top part and bottom part connected by a frame with two columns of support. The parts can move linearly in the test direction (see Fig. 2a). Each part is equipped with hydraulic, parallel grips that clamp the specimen. The top part of the testing device connects to the crosshead of the universal testing machine (Inspekt 10, Hegewald & Peschke, Nossen, Germany) and transmits the test force. The clamping pressure of the hydraulic grips varies depending on the material being tested and the expected compression strength. Due to the wide range of compressive strength across the different veneer orientations, the pressure was set between 60 bar (90-direction) and 200 bar (00-direction) to prevent slipping and damage to the specimen during testing. The testing speed was equivalent to a strain rate of 1% per minute. The applied load was measured with a 10 kN load cell. Prior to starting the test, a pre-force of 2 to 10 N was applied to the specimen.

The clear test length between the grips in the loading direction must be chosen so that stability failures, such as buckling, do not occur during testing. This length depends on the specimen’s thickness and was determined in preliminary tests (see Table 3). The clear test length was reduced until failure occurred due to a force drop (00-direction) or a defined strain limit (90-direction), not buckling. The goal was to select the largest possible clear test length to avoid the potential influence of clamping on strain measurement. Buckling of a specimen was defined as a deflection in the thickness direction of more than 5% of the specimen’s thickness. Since there was no force drop due to failure in the 90° direction, but rather a force plateau, a 2% strain in the load direction was defined as the test end criterion.

**Table 3:** Clear test length of compression test in dependence of material thickness and load direction.

Veneer thickness	00-direction	90-direction
1 mm	5 mm	3 mm
2 mm	10 mm	5 mm
2.5 mm	8..10 mm	5 mm
3 mm	15 mm	5 mm

The same stereo camera system (Aramis Adjustable 12 M, Carl Zeiss GOM Metrology, Braunschweig, Germany) used for tension tests was used for the strain measurement. The DIC measurement was always performed on the closed veneer side (without checks). Therefore, the samples were prepared using the same patterning technique described earlier for the tensile test within the AOI. With this system setup, the strain in the loading direction was measured according to the free clamping length, with a margin to the clamping jaws of 1 mm, and the transverse strain of the specimen was measured according to the specimen’s width. This resulted in a minimum measurement range of 1 mm in the loading direction at a minimum free clamping length of 3 mm in the 90-direction. It should be noted that the correct measurement of strain transverse to the loading direction is hindered by the clamping jaws. Reliable values are only yielded with a free clamping length of at least 10 mm^14^. Additionally, the out-of-plane deflection of the specimen in the thickness direction was monitored to check for buckling. Detailed information on the DIC setup used and its parameters is provided in Table 2.

### Shear test

**Fig. 3:**
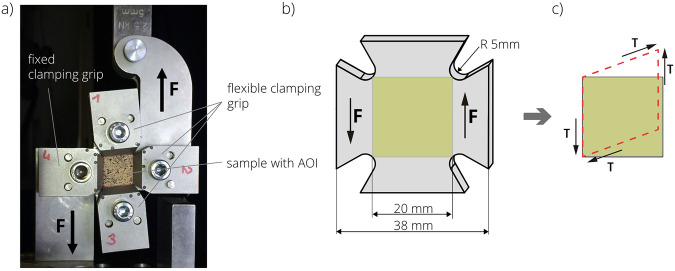
Shear test; (**a**) testing device; (**b**) sample dimension; (**c**) schematic shear deformation.

Figure 3 shows the shear frame test set-up and the specimen’s shape and dimensions. To measure the shear behavior, the uniaxial tension load is converted into shear force loads using the pivotally supported clamps of the shear frame test device. The clamp on the left side is fixed to the base plate which is also mounted to the testing machine. All other clamps are pivotally supported. The right clamp is linked to the crosshead of the testing machine via a bolt, which reduces out-of-plane forces and moments. This clamp can traverse in vertical direction. Thus, the shear frame is loaded and deformed by pure shear loads. This deformation is transferred to the specimen. The result is a shear stress state in the specimen whereby its geometry deforms from square to rhombic under quite uniform shear stresses throughout its cross-section (see Fig. 3c).

A square test specimen with recessed corners is held in the shear frame by four clamps bolted to the fixture. Shear stress is transferred to the specimen through frictional forces along the clamp surfaces. Preliminary tests determined an optimal tightening torque of 5 Nm for the screws. This prevents the specimen from slipping in the clamping fixture while avoiding indentation damage to the clamping surface. The same universal testing machine used for tension and compression tests was used for these tests. The testing speed was 0.4 mm/min, corresponding to a strain rate of about 1% per minute. The applied load was measured with a 10 kN load cell. Before starting the test, a pre-force of 5 N was applied to the specimen. The stereo camera system (Aramis Adjustable 12 M, Carl Zeiss GOM Metrology, Braunschweig, Germany) was used to measure the shear strain on the specimen surface. Detailed information on the DIC setup used and its parameters is provided in Table 2.

## Data Record

As described in the Methods section, data were collected from three primary experimental sources: uniaxial tensile test, uniaxial compression test, and shear test. Two datasets are available in the open-access data repository Zenodo: one for the HoMaba project^16^ and one for the HoBaCo project^17^.

Each dataset consists of two directories. The “01_Data” directory contains all the experimental data, organized into subdirectories and stored as a CSV text file for each test. The subdirectory structure varies by research project and is described below.

**Fig. 4:**
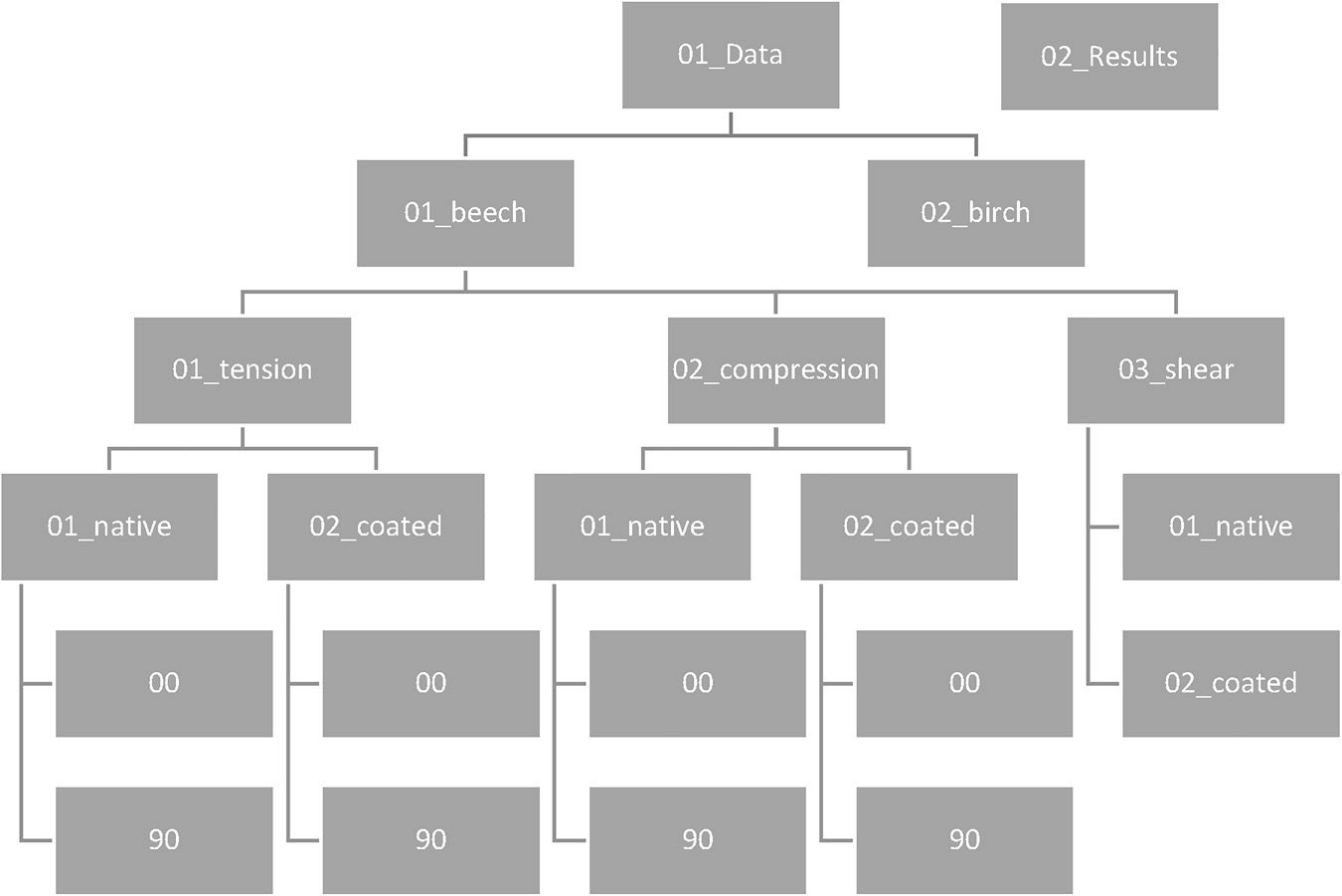
Directory structure for the HoMaba-project.

Figure 4 shows an overview of the directory structure for the HoMaba project. The first level distinguishes between the two wood species examined: beech and birch. The second level is organized by type of mechanical test (tension, compression, or shear). The third level subdivides the test data according to a possible surface coating with adhesive (native, meaning no coating; or coated). For tension and compression tests only, the final level classifies the fiber direction of the samples in relation to the loading direction (00 - parallel to the fiber direction and 90 - perpendicular to the fiber direction).

**Fig. 5:**
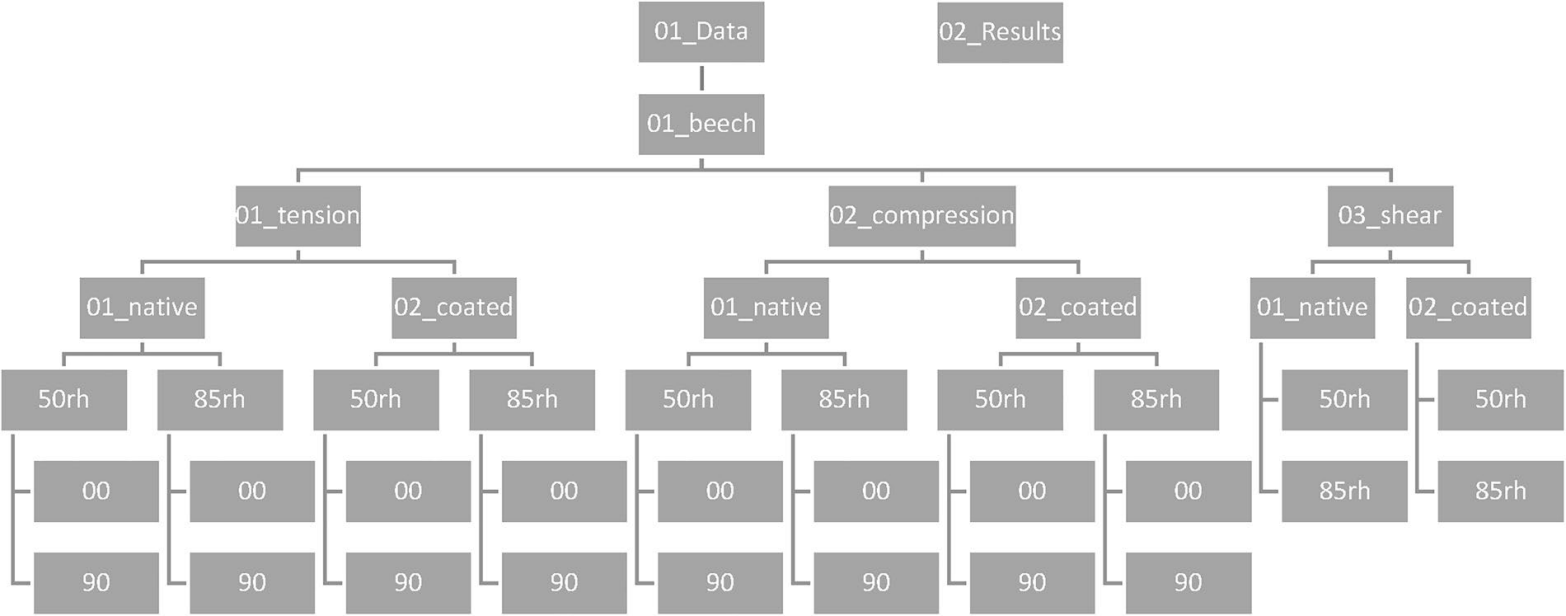
Directory structure for the HoBaCo-project.

The directory structure for the HoBaCo project is similar to that for the HoMaba project and is shown in Fig. 5. The difference is that an additional directory level has been inserted before the final level in order to distinguish the fiber direction of the test specimens. This level classifies the test conditions, particularly the relative humidity at which the test was performed (50rh–50% relative humidity and 85rh–85% relative humidity).

The data record for each test is saved as a CSV text file. The file header contains descriptions of the following test parameters:

- wood species;
- test type;
- test conditions include
  - force direction;
  - measuring field length;
  - measuring field width;
  - temperature;
  - relative humidity;
  - testing speed;

- specimen properties include
  - surface coating;
  - specimen length;
  - specimen width;
  - specimen thickness;
  - density;
  - moisture content.

Below the header are the measured stress-strain data, which have the following columns:

- stress in force direction (tension and shear with a positive sign and compression with a negative sign);
- strain in force direction (using AOI, except for tensile test in 90-direction measured by video extensometer);
- strain perpendicular to the force direction (for tension and compression only).

The “02_Results” directory for each dataset contains two CSV text files: one with the evaluated mechanical properties per test (results.csv) and one with the mean values per test series (results_mean.csv). The evaluated parameters are:

- Young’s modulus for tension and compression, and shear modulus for shear loading;
- Poisson’s ratio (for tension and compression only);
- ultimate stress at failure;
- ultimate strain at failure.

## Technical Validation

All measuring devices (the Inspekt 10 universal testing machine and the Aramis adjustable 12 M stereo camera system) were calibrated by their respective manufacturers.

To verify the applicability of the developed test methods, an exemplary round robin test was carried out for the tensile test. Eight independent testing institutes participated in this test and successfully demonstrated the comparable applicability of the developed test method using high-density fiberboard made of pine wood as a homogeneous wood-based material^10^.

To use the measured data as consistent quasi-static data, a uniform strain rate of 1% per minute was specified for the samples. This target speed was determined in preliminary tests because the available testing machine only has constant displacement control in millimeters per minute.

Prior to conducting the test series for tension, compression, and shear, preliminary tests were carried out to determine the optimal test parameters. This ensured that comparable experimental data could be recorded for later evaluation.

The recorded test data were used to evaluate the basic mechanical parameters, including Young’s modulus, Poisson’s ratio, and shear modulus. These values were determined within the elastic range of the stress-strain curve, between 15% and 35% of ultimate stress, using linear regression. These parameters were determined using the GOM Aramis system’s software. The coefficient of determination also served as a quality criterion for the data set. For the respective data record to be included in the repository, all defined parameters had to be determinable from the recorded test data and have a coefficient of determination greater than 0.8.

The goal was to obtain 20 valid results for each testing method and series using 25 prepared samples. The number of valid measurements is equal to the number of files saved within each measurement series of the datasets, as summarized in the “results_mean.csv” file.

## Usage Notes

The data in these datasets can be used for various purposes. First, other scientists can examine and evaluate the raw data from the measured stress-strain curves in accordance with their questions and objectives. On the other hand, the datasets for rotary-cut veneer of different thicknesses and wood species offer comprehensive, related material data and characteristic values that have not yet been published in their entirety in existing literature. These values can serve as a basis for comparison in future studies by other scientists. Additionally, the data can be useful for simulation purposes. When designing and optimizing structures with simulation tools, suitable material properties are required as input data, which these datasets provide.

## Data Availability

Data were deposited in the Zenodo open data repository and are available at the following 10.5281/zenodo.17201843 and 10.5281/zenodo.17208606.

## Code availability

No custom code was used.
